# Detailed kinetic and chemometric study of the cellulose thermal breakdown in artificially aged and non aged commercial paper. Different methods for computing activation energy as an assessment model in archaeometric applications

**DOI:** 10.1186/1752-153X-6-S2-S7

**Published:** 2012-05-02

**Authors:** Federico Marini, Mauro Tomassetti, Stefano Vecchio

**Affiliations:** 1 Department of Chemistry, Univesity of Rome “La Sapienza”, P.le Aldo Moro 5, I-00185 Rome, Italy; 2 Department S.B.A.I., University of Rome “La Sapienza”, Via del Castro Laurenziano 7, I-00161, Rome, Italy

## Abstract

**Background:**

The thermal oxidative degradation of aged and non aged cellulose samples of commercial paper was studied using thermogravimetry and derivative thermogravimetry under a forced air flow up to 800°C.

**Results:**

TG and DTG data were processed using two non-isothermal-based model-fitting methods and one based on linear least squares to calculate *E*_a_ trend values, measured as a function of artificially induced sample age. The *E*_a_ trends thus obtained were compared in order to assess their potential for yielding archaeometric curves. As the trends of first two methods show an inversion of the direction between non aged cellulose samples and artificially aged samples, while the third method does not, an in-depth study was carried out using a multilinearity assumption.

**Conclusions:**

The results are discussed and the outcomes indicate that the above cited inversion is real and not linked to the method. Additionally, it was evidenced that the number of points used for the estimation of linear least squares model parameters is of capital importance.

## Background

Several authors have demonstrated that of all the components contained in paper or wood it is cellulose in particular that is gradually oxidized with the age [1-4].

It was postulated that in the paper or wood samples respectively aged naturally or artificially, the state of the cellulose polymer chains is damaged by well-known oxidative, photo-oxidative and hydrolytic processes, which occur during ageing [2]. It was therefore attempted to correlate ageing, or the age, of the cellulosic samples with age-linked parameters that could be measured experimentally, if possible using relatively simple procedures and thus avoiding expensive apparatus [5-7].

Therefore, our group recently investigated the possibility of constructing curves, known as *archaeometric curves*, as a function of the ageing cellulose, paper or wood items [1,5,6].

In essence, it was endeavoured to detect any relationship between the breakdown of the cellulose and the physico–chemical parameters by which this could be evidenced. Consequently, the thermal oxidative degradation of non aged, or artificially aged, cellulose samples of commercial paper as such, or UV irradiated in a weatherometer, were studied using thermogravimetry (TG) and first-order derivative thermogravimetry (DTG) [8]. In this framework the activation energy (*E*_a_) values, which are related to cellulose fiber packing, seem to be more promising for the purpose of constructing archaeometric curves [9]. However, there appears to be no recommended literature method for calculating the E_a_ of the cellulose thermal degradation process. Thus, in a previous study [10], in order to obtain reliable activation energy values and to identify the method yielding the most reliable data, a comparison was made between the results obtained by applying three different methods for calculating the E_a_ of the oxidative thermal breakdown of the cellulose contained in several different samples of aged and non aged paper. In particular, two non isothermal model-fitting methods – the differential Arrhenius [9,11] and the integral Satava [9,12] methods - were applied and the results obtained compared with those determined using the Wyden-Widmann method [13]. All three methods are based on the linearization of suitable equations using the least squares method, from the slope of which the *E*_a_ values are determined. Nevertheless, a good correlation was found between *E*_a_ trends obtained using Arrhenius’ and Satava’s calculation methods and only partially with those obtained using Wyden-Widmann’s method.

The *E*_a_ trends obtained so far from TG/DTG data as a function of sample age for artificially aged extra-strong paper samples using the first two methods have not always been found to be completely linear. However, an inversion of the direction of these trends was found above all on going from fresh samples to aged samples, while the trends seem to be sufficiently linear whenever only samples of different artificial ageing times are considered.

For this purpose, from a previous study [10], it was found possible only to conclude that the cause of the inversion in the direction of the trend of *E*_a_ values observed in non aged cellulose samples, compared with aged samples, may be different and is often multiple in nature. For the time being it is not possible to conclude what the most plausible, or at least the most prevalent, reason might be that a degree of synergism possibly occurs also between several causes of this phenomenon. Furthermore, it must be determined to what extent the kinetic method used to calculate the *E*_a_ values actually affects the trends obtained. This point obviously calls for a more thorough investigation in order to to achieve full elucidation, which is precisely what has been attempted in the present note.

## Results and discussion

**Figure 1:**
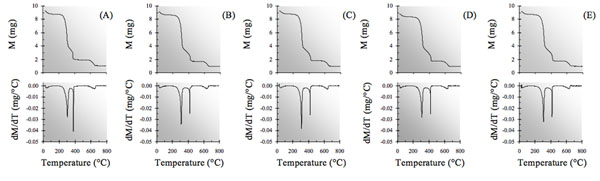
TG and DTG curves of extra-strong paper samples either non aged (A) or aged at the following ageing times: 100 h (B), 245 h (C), 500 h (D) and 750 h (E).

As stated above, a reasonable correlation was found in a previous paper [10] between the trends of E_a_ values obtained using Arrhenius’ and Satava’s methods and partly also from the trend found using the Wyden-Widmann least squares method (MLR) applied to the data obtained from the TG and DTG curves of artificially aged extra-strong paper shown in Fig 1, while some difficulty was found when the E_a_ value of cellulose thermal degradation of non aged paper was compared with that referring to the same process in non aged paper found using Arrhenius’ or Satava’s methods (the TG-DTG curve of which is also reported in Fig. 1).

**Figure 2:**
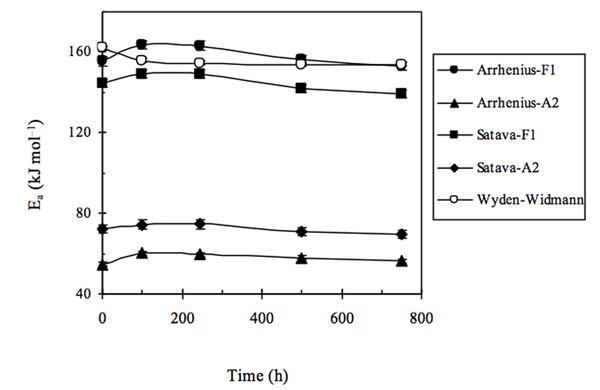
Activation energy (E**_a_**) trends of extra-strong paper samples either non aged or artificially aged for several ageing times using three different methods for processing TG - DTG data.

In the latter case, the representative *E*_a_ point was quite anomalous and differed as to the trends obtained (see Fig. 2), so that in the latter case non negligible differences over the entire trend of *E*_a_ values were found between the first two model-fitting methods and that obtained using Wyden-Widmann’s method.

In the interpretation of these outcomes, it was not clear to what extent they depended on the real differences in the physico-chemical characteristics of the cellulose in the non aged sample versus the aged samples and, conversely, to what extent the kinetic method used to calculate the *E*_a_ values actually affected the *E*_a_ trends obtained using the equations (6, 7 and 9) for the Arrhenius, Satava and Wyden-Widmann methods, respectively. In the present research Wyden-Widmann’s method, which is very often applied automatically using computer implemented software coupled with the thermogravimetric apparatus, was thoroughly tested. Initially, computation of Wyden-Widmann model parameters was also tried using a non-least squares iterative method based on continuous-valued genetic algorithms [14] but results statistically comparable to those ones deriving from the application of the MLR method were obtained, both when applied to the linearized version and when nonlinear solution was evaluated directly. However, as also discussed in the Experimental section, we observed how the estimated value of *E*_a_ can change significantly depending on the temperature range (and, as a consequence, on the number of data points) selected for use in the fitting procedure. Accordingly, the computation of model parameters in the Wyden and Widmann method was repeated by including a different number of data points in the fitting procedure in order to account for this observation.

**Figure 3:**
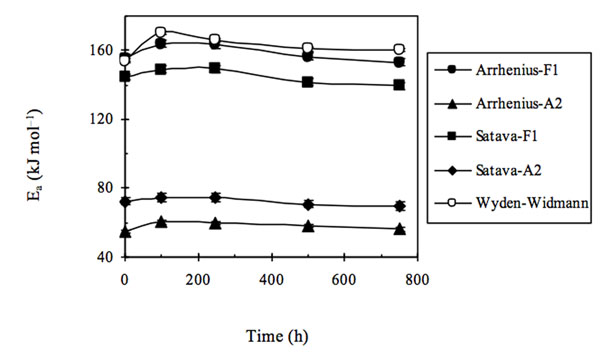
Activation energy (E_a_) trends of extra-strong paper samples either non aged or artificially aged for several ageing times using three different methods for processing TG - DTG data. Wyden–Widmann’s method was applied to a number of points > 60.

When examining the results obtained we realized that a reasonable correlation existed (see Fig. 3) between the trends obtained using the Arrhenius and Satava methods with that found by the Wyden-Widmann method when a sufficiently large number of experimental points was used (at least > 60).

**Figure 4:**
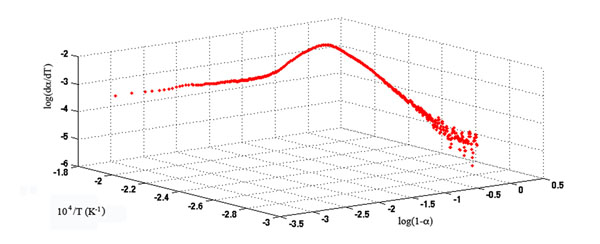
Representation of the TG/DTG data points for t = 250 h, in the linearized Wyden - Widmann space, showing the deviation from multi-linear trend.

**Figure 5:**
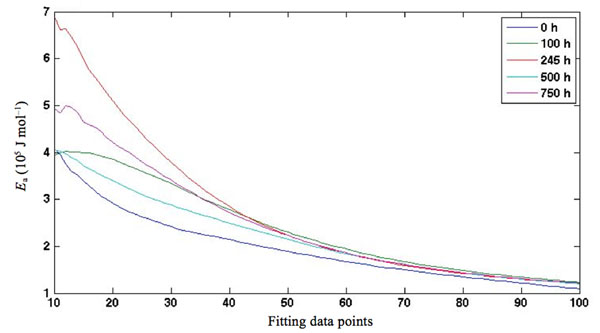
Kinetic modeling of “extra-strong” paper with Wyden and Widmann’s method: dependence of the estimated E_a_ value on the number of data points used for fitting. The reported number of data points is the number of points chosen symmetrically to the left and right of the temperature corresponding to the minimum in the DTG curve; hence 20 points means a 41 point interval centered with respect to T_min_.

Indeed, a thorough investigation, as shown in Fig. 4, revealed that the data corresponding to the TG/DTG curves of extra-strong paper, after a logarithmic transformation (see equation (9)), fit the multilinearity assumption only over a limited temperature range. Hence, the quality of the fitting process is highly dependent on the number of points used to compute the regression model, as shown in Fig. 5.

In particular, when Wyden - Widmann’s method is used, a trend similar to those found using Satava’s and Arrhenius’ methods is obtained only when a number of points lying between 62 and 100, before and after the DTG peak are processed in the fitting. In practice it was thus possible to conclude that: (i) the most correct E_a_ trend, as a function of the irradiation time in the weatherometer, seems to be the one obtained using Arrhenius’ or Satava’s methods, based on the identification of the model function that best fits the experimental trend of the DTG peak value - in practice the type of reaction mechanism [11,12]. However, a sufficiently correct *E*_a_ trend (i.e. in sufficient agreement with the Satava and Wyden – Widmann trend) may be obtained also by applying the Wyden-Widmann method but using only an appropriate number of points right and left of the DTG peak, (ii) It therefore seems that the inversion of trend found on going from the non aged extra-strong paper sample to the artificially aged samples is real and linked to physical – chemical differences between the non aged and aged paper matrixes (at least for the *E*_a_ values referring to the thermal degradation of the commercial extra-strong paper studied in the present research), (iii) When the results of *E*_a_ values obtained by applying all three methods are compared in this way (see Fig. 3), it seems that the most probable *E*_a_ values lie in the range of 150-180 kJ mol^–1^ and that the fitting procedure indicates that the most suitable mechanism of all the functions reported in Table 1 is the F1 function (first-order mechanism).

**Table 1:** Model functions considered in this study along with their expressions.

Model mechanism	Symbol	Mathematical expressions
First-order decay (*n*=1)	F1	(1–*α*)
Second-order decay (*n*=2)	F2	(1– *α*)^2^
Third-order decay (*n*=3)	F3	0.5 · (1– *α*)^3^
Avrami-Erofeev (*n*=2)	A2	2 · (1– *α*) · [–ln(1– *α*)]^0.5^

**Figure 6:**
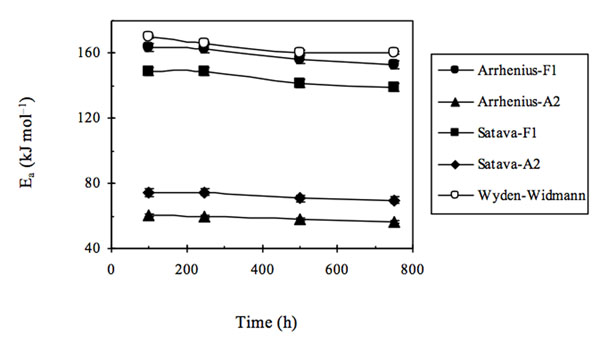
Activation energy (E_a_) trends of extra-strong paper samples artificially aged only for several ageing times using three different methods for processing TG - DTG data. Wyden–Widmann’s method was applied to a number of points > 60.

Furthermore, if the same procedures to calculate *E*_a_, as those used in the three methods examined are repeated for values referring to all the extra-strong paper samples tested at different irradiation times, excluding only the non irradiated sample and using all three methods considered alternately, it may be concluded (see Fig. 6) that, in this case, the function F1 produces the expected quasi monotonically decreasing trend and at the same time E_a_ values having an order of magnitude equal to those previously found and that also Wyden-Widmann’s method, again using a number of experimental points approximately lying between 40 and 60, in this case produces a trend in *E*_a_ values very similar to those of the two Model Fitting methods.

**Figure 7:**
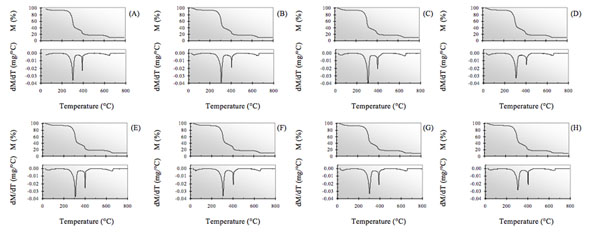
TG curve and DTG curves of “Fabriano” paper samples, either non aged (A) or aged at the following ageing times: 25 h (B), 40 h (C), 50 h (D), 75 h (E), 100 h (F), 150 h (G) and 200 h (H).

Once again this seems to indicate that the anomalous value obtained for the non irradiated sample does not depend on the method used to calculate *E*_a_, but on physico-chemical differences inherent in the sample itself vis-à-vis the irradiated samples. Further confirmation of this conclusion was obtained by observing the *E*_a_ trends obtained using each of the three calculation methods described herein but referring to the TG-DTG curves shown in Fig. 7 recorded for another type of commercial Italian paper - “Fabriano”, both artificially aged and non aged.

**Figure 8:**
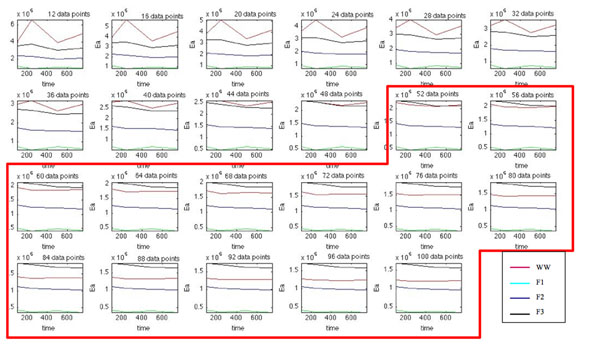
Kinetic modeling of “Fabriano” paper using Wyden-Widmann’s and Arrhenius’ (using functions F1, F2 and F3) approaches: representation of the computed E_a_ time decay trends as a function of the number of data points used for fitting. As in Figure 5, the reported number of data points is the number of points chosen symmetrically to the left and right of the temperature corresponding to the minimum in the DTG curve.

As shown in Fig. 8 good agreement in this case is found among the *E*_a_ value trends obtained using the customary three calculation methods only when, in the case of the Wyden-Widmann method, the number of values processed around the DTG peak is ≥ about 50. Here the anomaly observed for the *E*_a_ value of the non irradiated sample of extra-strong paper does not occur. This amounts to a definitive confirmation that this anomaly is to be attributed to the non irradiated sample and not to the computation method used.

**Figure 9:**
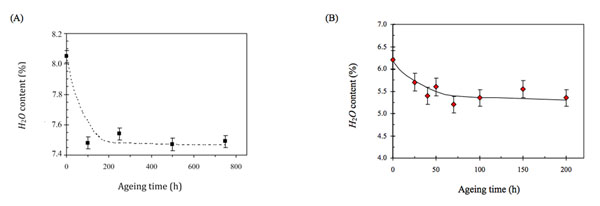
Trend of the percentage of water loss contained: (A) in the “extra-strong”, (B) in the “Fabriano” paper samples, as a function of the ageing time.

Lastly, the decrease in the total water content in aged, in comparison with non aged, paper samples, detected by us using TG measurements (see Fig. 9) appears to be related to some extent to the decrease in microcrystalline domains in the paper samples and to the increase in the amorphous domains which seems to occur with increasing paper sample irradiation time. Our thermogravimetric method findings actually seem to be in good agreement with the results of NMR studies on several paper samples [15-17]. These studies demonstrated that water is wholly bound to the cellulose lattice, but only semi-crystalline fibrous cellulose is rich in water, while the amorphous variety has a low water content. On the other hand, comparative NMR measurement also of the total water amount in paper samples revealed a different degree of degradation linked to total water variation. It was thus possible to conclude that the loss of water is associated with increasing cellulose degradation of polymorphs, which, in turn, may be associated with the increase in the amorphous fraction. Moreover, the results of NMR measurements agree with those obtained using the SANS method [18], that is, in both cases clusters (or microdomains) in which the highest concentration of water contained in the aged paper samples were detected.

## Experimental

### Samples and apparatus

Paper ageing was achieved by photoirradiation at λ = 310 nm in a weatherometer (Model QUV/spray Q-Panel LAB-Products) for up to 750 hours. The apparatus was equipped with 8 mercury lamps (40 W each) able to simulate sunlight perfectly up to 370 nm.

Samples of extra-strong paper for photocopiers and laser printers (white paper for office photocopiers, Copy blu, Code 13092 - 6IN - format 21x29.7 cm, A4, basic weight 80 g m^–2^) were studied either as such or artificially aged in a weatherometer for different ageing periods up to a maximum of 750 h.

The TG/DTG measurements were carried out on a Mettler 50 thermobalance coupled with a Mettler TG 10-TA processor system and to a Swiss dot matrix printer.

For the purpose of performing thermogravimetric experiments, from each extra-strong paper sample 5 small circular disks (5.0±0.1 mm in diameter) were cut out, each weighing 1.7±0.1 mg. The diskettes were then placed in a special cylindrical alumina crucible, 0.7 cm in diameter, and carefully compressed using a small piston before being subjected to TG analysis under a forced air flow (0.1 dm^3^ min^–1^) at a heating rate of 10 °C min^–1^.

Another typical and well known commercially available Italian paper (“Fabriano” paper), both non aged or artificially aged in a weatherometer, was also analysed. In this case all the paper samples were minced finely before analysis. The size of individual paper fragments was of the order of 1.0 ± 0.2 mm^2^ on average.

In the course of artificial ageing in the weatherometer, Fabriano paper samples were taken at several irradiation times up to a maximum of 200 hours.

## Kinetic theory

### Determination of activation energy using the Arrhenius and Satava model-fitting procedure

The thermal breakdown of the cellulose contained in the paper and wood samples was studied by treating the TG/DTG data referring to this process. The kinetic study of the reactions undergone by a compound in the solid state is generally based on the following relation:(1)

where *α* is the degree of conversion, calculated according to the following equation:(2)

where *m*_i_, and *m*_f_ are the initial and final sample mass, while *m*_T_ is the sample mass at a given temperature *T*; *k* is the process kinetic constant and *f*(*α*) is a function defined by different mathematical expressions depending on the type of reaction mechanism [19,20]. The explicit dependence of the reaction constant on temperature is obtained by expressing the kinetic constant *k* using the Arrhenius equation:(3)

which, when included in Eq. (1), gives:(4)

in which *A* (pre-exponential factor) and *E*_a_ (activation energy), are the Arrhenius parameters and *R* is the universal gas constant. When the sample is subjected to controlled heating at a constant rate *β*, where *β* = d*T*/d*t*, the explicit dependence of reaction rate d*α*/d*t* on time in equation (4) is eliminated, as:

and so:(5)

Multiplying both sides of equation (5) by *β* · *f*(*α*)^–1^ and calculating the natural logarithms, the following equation is obtained:(6)

which is used in the first (differential) method, also called the Arrhenius method [9,11].

The values of *α* were also inserted in the integral function expression *g*(*α*), (where ) and used in the following Satava equation [9,12] as modified by Doyle [21] and Sestak [22]:(7)

In the two methods used, the left-hand side of equations (6) and (7) is assumed to be linear with respect to the reciprocal of the temperature (1/*T*) only for the functions *f*(*α*) and *g*(*α*) which represent the best mechanism for the reaction considered. From the slope and intercept of the regression straight lines described in equations (6) and (7) the values of the activation energy and the pre-exponential factor may be calculated. The differential and integral *f*(*α*) and *g*(*α*) functions that best fit the reaction mechanism were chosen from among those reported in literature [23] on the basis of experimental parameters referring to the shapes of DTG peaks related to the thermal oxidative process of cellulose contained in the wood or paper samples, as described in detail in previous papers [9,22]. The model functions reported in literature [21] and selected in previous papers for similar cellulosic samples [10] are denoted by the symbols A (Avrami-Erofeev models) and F (*n*^th^-order decay models).

### Determination of activation energy using the Wyden-Widmann method

Using this method the activation energy values were always obtained starting from the TG and DTG data, which were however processed by means of the multiple linear regression method proposed by Wyden-Widmann [13]. Also using this method the activation energy *E*_a_ of the cellulose decomposition step was calculated on the basis of the values derived from a single thermogravimetric experiment carried out at a constant heating rate, using the *n*^th^ order multiple linear regression method and adopting the following Arrhenius type equation:(8)

where *n* is the order of reaction.

By applying the least squares method, the sum of the squares of the differences between the values of d*α*/d*t* calculated by equation (8) and those derived using TG/DTG measurements (taking into account that *α* is calculated using equation (2) and d*α*/d*t* = (d*m*/d*t*) /Δ*m*_tot_ where d*m*/d*t* is the rate of mass loss and Δ*m*_tot_= *m*_f_–*m*_i_) attains its minimum value for given values of *A*, *n* and *E*_a_, which are thereby identified. In particular, the value of the activation energy *E*_a_ is thus determined.

### Further investigation: equation for multilinearity assumption

The core of Wyden and Widmann’s method [13] for estimating the kinetic parameters in equation (8) is the linearization of the rate law using a logarithmic transform leading to the expression:(9)

The left-hand side of equation (9) is linear with respect to both the reciprocal temperature and log(1-*α*). Therefore, it is customary for the values of the kinetic parameters to be computed following a multiple linear regression (MLR) approach. However, when the values of log(d*α*/dt), *T* and log(1-*α*) are obtained from the TG/DTG curves according to equation (9) and log(d*α*/dt) is plotted against (1/*T*) and log(1-*α*), not all the fitted data points are found to lie on the same plane, as is assumed in the same equation. Therefore, in order to obtain a reliable estimation of kinetic parameters, a suitable number of data points, sufficient to give an accurate fit but not so many as to include deviations from the linearity assumption, must be considered. Accordingly, in this investigation the effect of the number of data points to be used for computing model parameters in Wyden-Widmann’s approach on the quality of the final solution was also studied. This goal was obtained for each TG/DTG curve by computing MLR models based on an increasing number of data points from 21 to 201, symmetrically distributed around the temperature, corresponding to the minimum in DTG cellulose peak.

## Conclusions

It may be concluded that: (i) the most correct *E*_a_ trend, as a function of the irradiation time in the weatherometer, seems to be the one obtained using the Satava and Arrhenius methods to identify the model function that best fits the experimental trend of the DTG peak values, (ii) A sufficiently correct trend may be obtained also by applying the Wyden–Widmann method but only using a suitable number of points right and left of the DTG peak, (iii) It is possible to choose the appropriate number of points in Wyden-Widmann’s approach by taking into account the multilinearity assumption and observing the temperature interval over which it is followed, (iv) It therefore seems that the inversion of trend found on going from the non aged extra-strong paper sample to the artificially aged samples is real and linked to the non aged paper sample and not to the method used to compute the *E*_a_ values, (iv) the choice of the best functional form (in the case of model fitting methods) and of the number of points to use for the computation of the regression parameters in the case of Wyden-Widmann’s method, is of capital importance for a correct final result.

## Competing interests

The authors declare that they have no competing interests.

## Authors' contributions

FM performed chemometric data analysis using kinetic data processed by TG. SV processed TG/DTG data according to model-fitting methods and provided the related kinetic parameters. MT carried out TG/DTG measurements, coordinated the study. MT and SV edited the text and prepared the final draft of the paper. All the authors have read and approved the final manuscript.
